# Surgical Triumph Over Huge Nontraumatic Myositis Ossificans of the Gluteal Region in an Epileptic Patient With History of Stroke: A Case Report

**DOI:** 10.7759/cureus.60294

**Published:** 2024-05-14

**Authors:** Mainak Roy, Deepanjan Das, Suhas Aradhya Bhikshavarthi Math, Samir Dwidmuthe, Vivek Tiwari

**Affiliations:** 1 Orthopaedics, All India Institute of Medical Sciences, Nagpur, IND; 2 Orthopaedic Surgery, All India Institute of Medical Sciences, Nagpur, IND; 3 Department of Orthopaedics, Apollo Sage Hospital, Bhopal, IND

**Keywords:** stroke history, epileptic patient, hip pain, surgical excision, nontraumatic myositis ossificans

## Abstract

Myositis ossificans (MO) is a benign condition where bone forms within muscles due to increased activity of the periarticular tissues. Trauma is the most common cause. Nontraumatic MO is exceedingly rare. We present a rare instance of nontraumatic MO affecting the hip in a 32-year-old patient. The patient had a known case of seizure disorder and also had a history of a cerebrovascular accident (CVA). Despite the absence of trauma or known predisposing factors, the patient developed a sizable mass in the left hip, causing pain and restricted range of motion (ROM). Surgical excision of the mass was successful, resulting in complete removal and subsequent improvement in hip function and pain relief during postoperative recovery. Histopathological examination confirmed the diagnosis of MO. The patient's ROM normalized, and there were no signs of recurrence at the one-year follow-up. This case highlights the importance of recognizing MO in hip pain cases without trauma. Timely surgery through the approach described effectively removes the mass, preventing recurrence without compromising vital structures. It showcases a successful multidisciplinary approach for rare musculoskeletal conditions, offering valuable insights into similar cases.

## Introduction

Myositis ossificans (MO) is a benign condition where bone forms within muscles due to increased activity of the periarticular tissues [1]. It can occur in various parts of the body, with the hip, elbow, and wrist being particularly susceptible due to their vulnerability to injury [2]. Trauma is the most common cause of MO [3]. Nontraumatic MO is exceedingly rare. It typically does not require aggressive treatment since it is self-limiting. However, surgical removal might be recommended if MO affects joint movement, causes nerve or blood vessel compression, or is particularly large or painful [4]. Nontraumatic MO is a rare entity. This paper presents a rare case of nontraumatic MO of the hip in a patient with a previous history of stroke.

## Case presentation

A 32-year-old male patient presented with a history of pain and range of motion (ROM) restriction of the left hip. He also complained of a mass in the left hip for the past year. There was no history of trauma or any other similar diseases in the family. Notably, the patient had a known case of seizure disorder and was on medications for the last three years. He also had a history of stroke two years back, which was managed conservatively elsewhere with full recovery after six months of treatment. No related documents were available. The patient had an antalgic gait; 10 degrees of flexion deformity was noted in the affected hip with a ROM painfully restricted to 10-70 degrees of flexion, 10 degrees of internal rotation, 20 degrees of external rotation, 20 degrees of abduction, and 10 degrees of adduction. Tenderness was noted at the left groin and the left hip region. A bony hard mass was also palpable in the left hip and groin region. Neurological examination was within normal limits.

**Figure 1:**
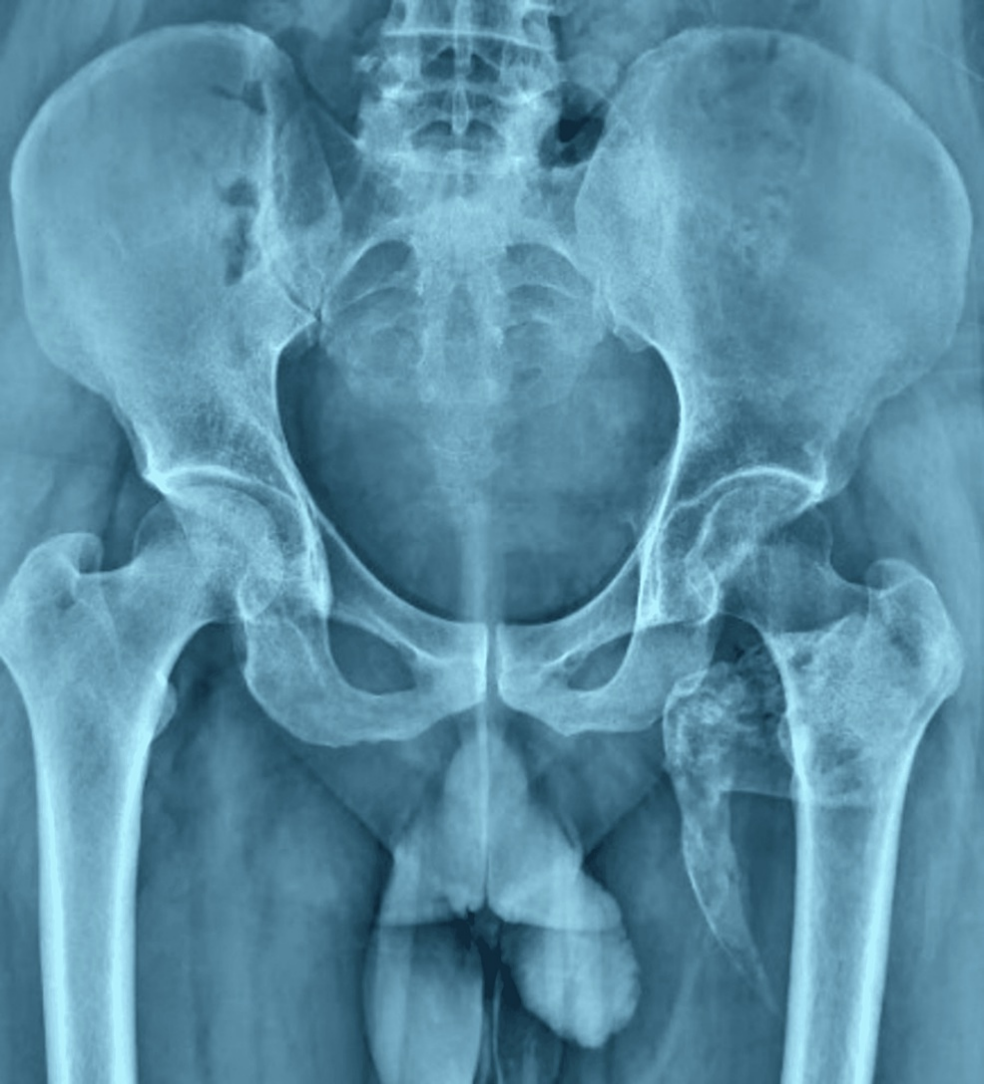
Pre-operative radiograph showing a radiopaque irregular mass adjacent to the proximal femur

X-ray imaging showed a mass visible as a radiopaque area near the proximal femur (Figure 1).

**Figure 2:**
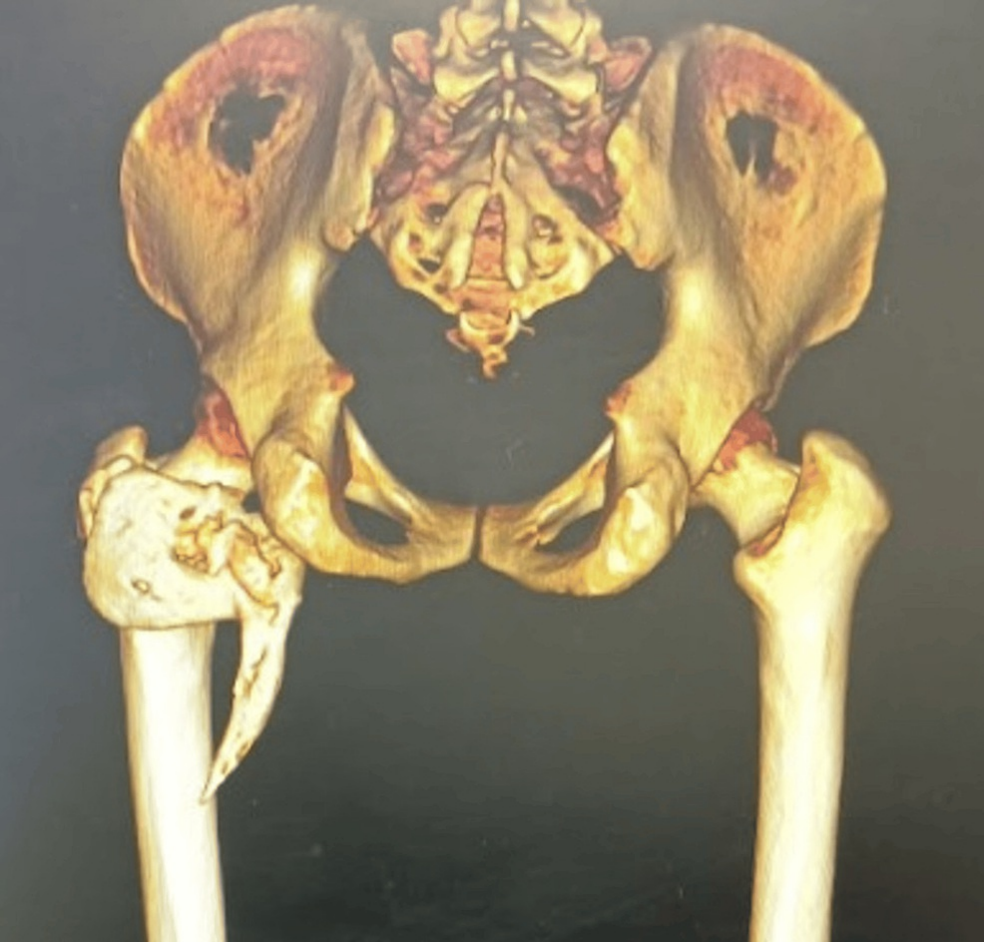
Pre-operative CT scan showing an irregular bony mass extending from gluteal region to subtrochanteric area of femur

CT scan showed an L-shaped osseous mass extending from behind the pelvis and basicervical region of the femur to below the lesser trochanter with a pointed spike extending to the proximal region of the femoral shaft (Figure 2). The medial extent of the mass was almost until the ischial tuberosity and the lateral extent was until the greater trochanter region.

**Figure 3:**
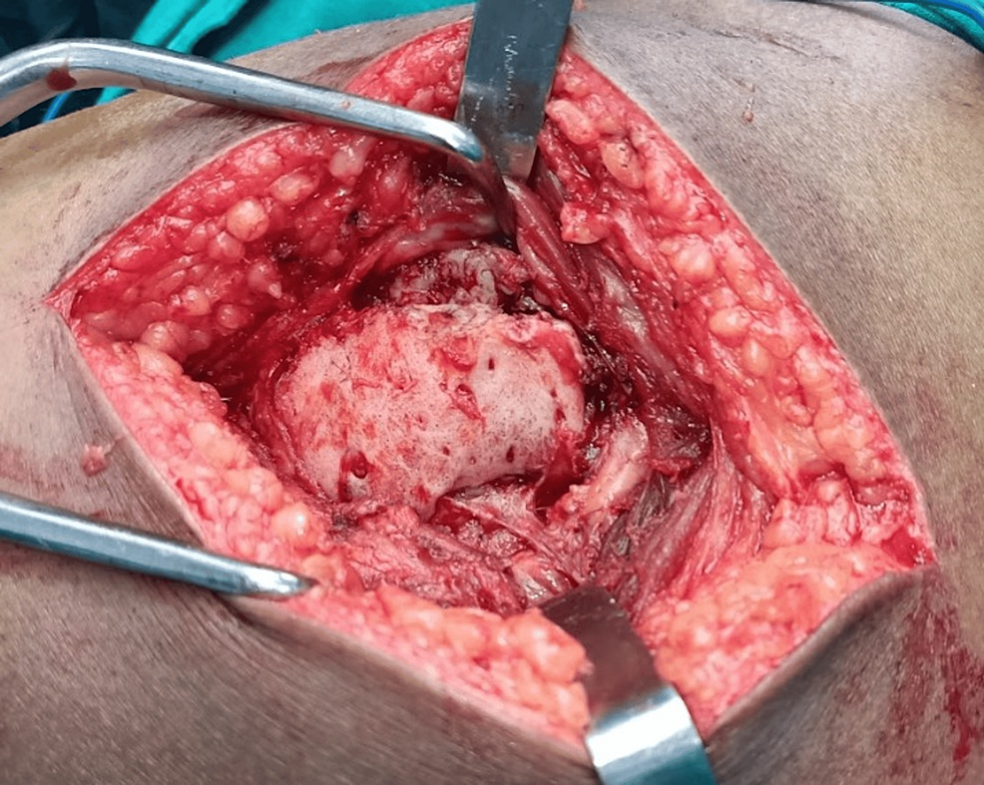
Intra-operative image showing a bony hard mass surrounded by a periosteum-like capsule within the substance of the gluteus maximus extending into the submuscular plane

The patient was planned for an excision of the mass with proper informed consent. The patient was taken in a right lateral position and a standard posterior approach to the hip was taken. After routine soft tissue dissection, a bony hard mass surrounded by a periosteum like capsule was found within the substance of the gluteus maximus extending into the submuscular plane (Figure 3).

**Figure 4:**
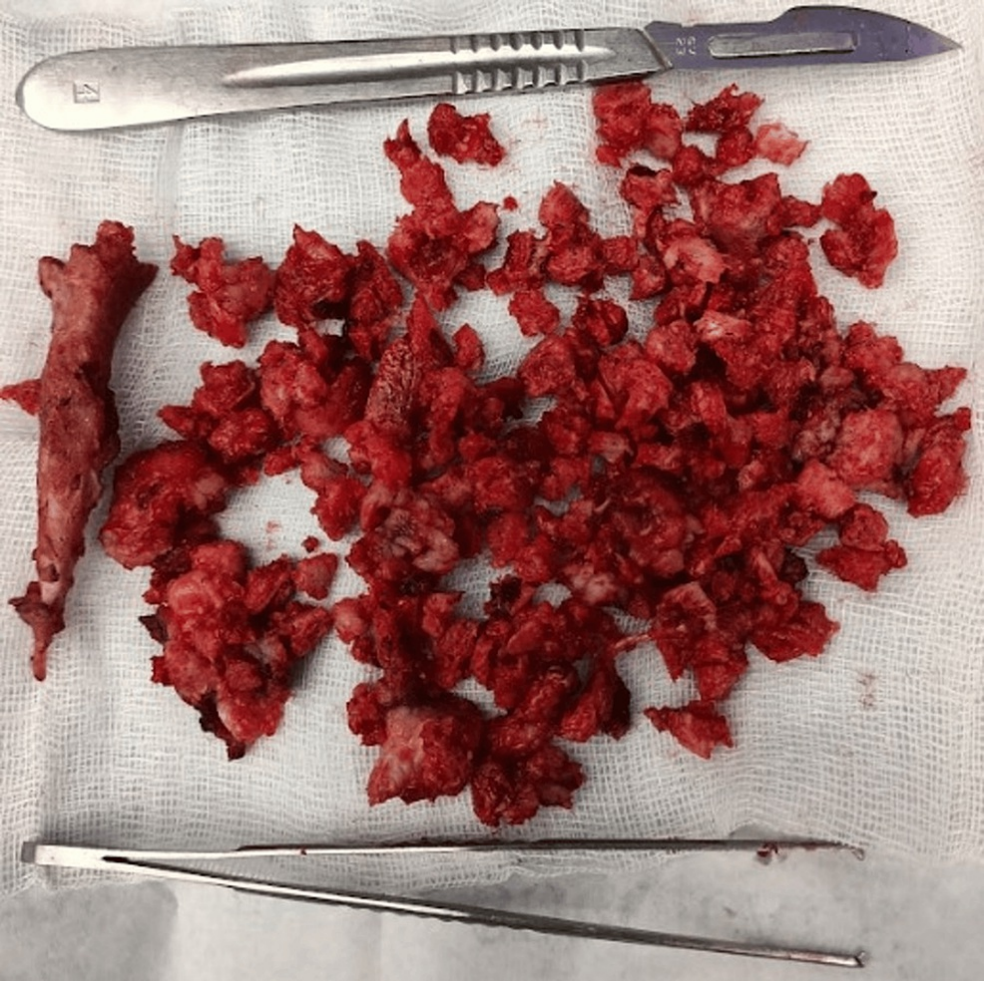
Small fragments of the excised bony mass

The sciatic nerve was uninvolved despite the lesion being so close to the nerve. The mass was excised in pieces after breaking it down into small fragments using a bone nibbler and osteotomes (Figure 4).

**Figure 5:**
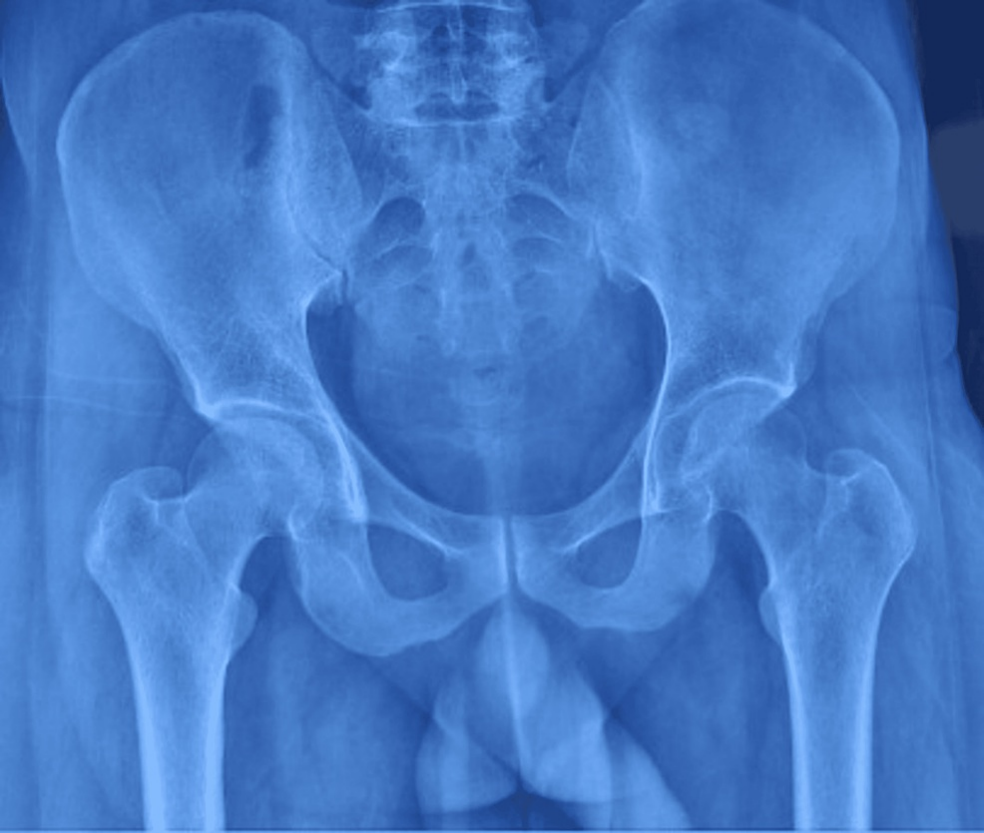
Post-operative radiograph showing complete excision of the MO mass MO: Myositis ossificans

The approach allowed for excellent visualization and complete removal of the mass, at the same time protecting the vital structures around the area. The distally extending spike of the mass measured around 15 cm in length. The procedure was uneventful. The patient was started on hip ROM exercises and full weight bearing from the day after the surgery. Post-operative recovery was fair, with a significant reduction in pain and improved ROM of the hip. Post-operative radiographs showed complete excision of the MO (Figure 5).

**Figure 6:**
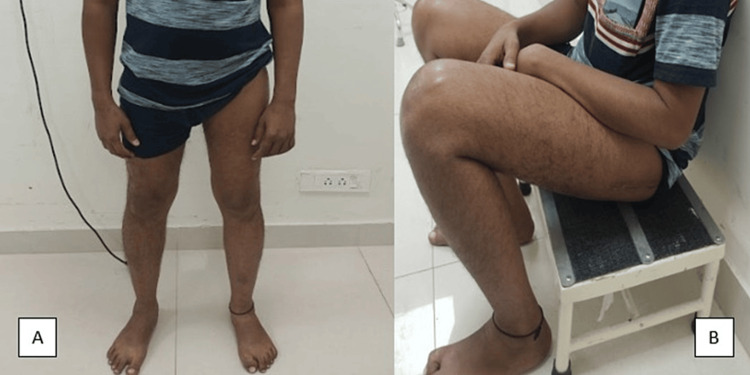
Clinical profile at the one-year follow-up showing (A) normal hip extension during standing and (B) normal hip flexion during sitting position

Histopathological examination of the lesion revealed mature bone tissue, confirming the diagnosis of MO. The patient was started on indomethacin 75 mg twice daily post-operatively, which was continued until six weeks post-surgery. The patient was also treated with a single dose of radiotherapy (RT) with 800 cGy. At one year follow-up, the patient was pain-free with a normal gait and with no hindrance in doing daily living activities. Hip ROM had also normalized with 120 degrees of flexion possible, along with 10 degrees of extension, 30 degrees of internal rotation, 30 degrees of external rotation, 30 degrees of abduction, and 30 degrees of adduction (Figure 6). No clinical or radiological signs of recurrence were evident.

## Discussion

MO is a benign condition characterized by the formation of well-differentiated bone within muscle tissue. It is a rare condition, with a prevalence of less than one per million [1]. Trauma is the leading cause of MO [3]. It is believed that following significant trauma, tissue damage or bleeding triggers an excessive response from blood vessels and fibroblasts, leading to the formation of bone within soft tissues [4]. Underlying conditions such as tabes dorsalis, syringomyelia, poliomyelitis, paraplegia, tetanus, and haemophilia may also predispose individuals to MO. It can occur even with passive ROM exercises. Rarely, burns, infections, and drug abuse have also been associated with the condition [5-7]. Nontraumatic MO is an extremely rare entity.

The case was unique as the patient developed such a significantly large mass despite having no history of trauma or any other underlying predisposing condition. The only history the patient had that can be linked to the occurrence is a history of seizure disorder and cerebrovascular accident (CVA), the exact underlying mechanisms of which remain incompletely understood.

Some evidence supports a humoral mechanism that accelerates fracture healing, leading to increased bone formation. Patients with central nervous system injuries often have elevated levels of certain circulating growth factors, such as insulin-like growth factor II, platelet-derived growth factor, interleukin-1, and interleukin-6, in their serum. Additionally, there may be a potential role of specific neurotransmitters, such as leptin, in regulating bone metabolism through interactions with the hypothalamus and sympathetic nervous system. All in all, multiple genes may be involved [8].

Histologically, collagen-producing cells are typically found at the centre. Surrounding this central area, there is increased osteoblastic activity, leading to the formation of immature bone. In the outermost region, lamellar bone is present, representing more mature bone tissue [9]. MO is usually self-limiting [6]. The decision to operate was prompted by the patient’s severe pain, restricted ROM, associated difficulty in mobilization, and decreased functional ability.

RT has been observed to reduce the size of the mass and promote its maturation in certain cases. A single low-dose RT treatment has been commonly utilized and shown to be highly effective in many instances. Successful RT treatment combined with an indomethacin protocol has also been reported [10]. The use of nonsteroidal anti-inflammatory drugs (NSAIDs) is attributed to their ability to systemically inhibit prostaglandins, as indicated by certain circulating humoral factors [11]. While the optimal timing for excision of ossification remains uncertain, delaying the procedure excessively can result in suboptimal functional recovery due to pathological changes induced by prolonged immobilization [12]. Joint surface narrowing, atrophy, and severe osteoarthritic changes in cases of prolonged immobilization can be seen, stimulating fibro-fatty proliferation and fibrous ankylosis [13].

At most recent follow-up visits, the patient demonstrated substantial improvement in the ROM around the affected hip. Moreover, he did not demonstrate any signs of recurrence, whether clinically or radiographically. The approach highlights an effective way to completely excise such a large mass without compromising surrounding neurovascular structures, reducing the morbidity for the patient and preventing recurrence, leading to a satisfactory outcome.

## Conclusions

This case report highlights a rare occurrence of nontraumatic MO of the hip in a patient with a history of stroke. Despite the absence of trauma or other predisposing conditions, the patient developed a significantly large mass in the hip region. Surgical excision of the mass was performed successfully, with complete removal confirmed on post-operative imaging and histopathological examination. Post-operative rehabilitation, including NSAID and physical therapies, resulted in significant improvement in hip ROM and pain relief. The patient demonstrated no signs of recurrence at the one-year follow-up, indicating a favourable outcome. This case underscores the importance of considering MO as a differential diagnosis in patients presenting with hip pain and restricted ROM, even in the absence of trauma. This case also highlights the effectiveness of timely surgical intervention in achieving complete excision and preventing recurrence.
